# Soluble Urokinase-Type Plasminogen Activator Receptor (suPAR) Is a Biomarker Associated with Left Ventricular Hypertrophy in the Elderly, Specifically in Women

**DOI:** 10.3390/jcm12093290

**Published:** 2023-05-05

**Authors:** Rafał Nikodem Wlazeł, Agnieszka Guligowska, Zuzanna Chrząstek, Tomasz Kostka, Anna Jegier, Iwona Szadkowska

**Affiliations:** 1 Department of Laboratory Diagnostics and Clinical Biochemistry, Medical University of Lodz, Pomorska 251, 92-213 Lodz, Poland; rafal.wlazel@umed.lodz.pl; 2 Department of Geriatrics, Healthy Ageing Research Centre, Medical University of Lodz, Plac Hallera 1, 90-647 Lodz, Poland; 3 Department of Sports Medicine, Medical University of Lodz, Pomorska 251, 92-213 Lodz, Poland

**Keywords:** biomarker, soluble urokinase-type plasminogen activator receptor, suPAR, left ventricular hypertrophy, cardiac remodeling, echocardiogram, aging

## Abstract

Left ventricular hypertrophy (LVH) may result in the development of heart failure, which is widespread among people of advanced age. The pathophysiology of LVH is complex and its biochemical pathways are not fully understood in this group. Elevated soluble urokinase-type plasminogen activator receptor (suPAR), a biomarker of immune activation, including fibrosis, reflects subclinical organ damage in systematic diseases. The present study assesses the clinical role of suPAR measurement in determination of LVH-associated cardiac disorders in the elderly. The studied population consisted of 238 individuals aged 76–91 years; of these, 139 (58%) were diagnosed with LVH. Serum biomarkers measurement (suPAR, troponin T, NT-proBNP and CRP) and echocardiography were performed in all subjects. The suPAR level was significantly higher in the LVH group (4.01 vs. 3.82 ng/mL, *p* = 0.033) and correlated with the parameters of cardiac diastolic function. Stepwise logistic regression found suPAR level (OR = 1.55, *p* = 0.016), BMI (OR = 1.17, *p* = 0.0003) and hypertension (OR = 2.42, *p* = 0.046) to be independently associated with LVH in women. In men, the strongest predictors of LVH were hypertension (OR = 7.52, *p* = 0.014) and BMI (OR = 1.42, *p* = 0.032). The observations indicate suPAR as a promising marker reflecting LVH, especially in women at advanced age, independent of age-associated cardiac remodeling.

## 1. Introduction

The incidence of cardiovascular diseases (CVD) such as hypertension (HT), coronary artery disease (CAD) or heart failure (HF) rises significantly with age, affecting the majority of the elderly population. HT and CAD are the main causes of HF, which occurs in more than 10% of the people of advanced age [1,2]

Chronically elevated blood pressure that leads to pathological left ventricular hypertrophy (LVH) results mainly in diastolic dysfunction of the heart muscle and development of HF with preserved ejection fraction (HFpEF). On the other hand, myocardial ischemia is the main cause of HF with reduced ejection fraction (HFrEF). Regardless of the primary etiology of HF, fibrotic processes are indicated as the main cause underlying the cardiac damage [3,4]. In the elderly, physiological organ changes overlap those induced by comorbidities. The typical age-related cardiac remodeling includes the increase of left ventricular (LV) wall thickness as well as the decrease of LV dimensions and volumes, usually followed by the deterioration of LV diastolic function [5,6,7]. The coexistence of HT can exacerbate the above changes, and the differentiation between pathological and physiological hypertrophy on advanced age is not obvious. Furthermore, various mechanisms may underlie the unfavorable cardiac remodeling. 

The measurement of biochemical biomarkers reflecting distinct pathways may help in better understanding the clinical condition, as well as in prognosis. In this use, cardiac troponins and B-type natriuretic peptide remain of high interest, reflecting structural and functional abnormalities of the myocardium [8]. Nevertheless, the pathophysiology of LVH is complex, its biochemical pathways remain not fully understood and the interpretation of biomarkers’ pattern and its changes remain a challenge in the context of both diagnosis and prediction.

Soluble urokinase-type plasminogen activator receptor (suPAR) is a protein described as an independent prediction marker of the wide spectrum of conditions associated with chronic inflammation and immune system activation. Its elevated level is observed in individuals with chronic infections, and it also reflects subclinical organ damage in the systemic diseases [9,10]. Its clinical usefulness in risk stratification was described in acute care patients, in type 2 diabetes mellitus, chronic kidney disease and HF [11,12,13,14,15]. The protein is released from the membrane of immunologically activated cells (i.e., monocytes, macrophages, activated T-cells, fibroblasts, smooth muscle cells or endothelial cells, megakaryocytes and certain tumour cells) and is involved in pathways such as cell proliferation, migration and adhesion, fibrosis, atherosclerosis and many other immunological-related conditions [16,17]. In the general population, the suPAR level is higher in women and its concentration increases with age. Though it is still unknown how suPAR participates in the disease process, its association with CVD and ability to add to prognostic information in current risk stratification strategies of the patients seem to be unquestionable [18,19].

We have attempted to assess the clinical role of suPAR determination in the context of its usefulness in detecting disorders of cardiac structure and function in elderly patients. The suPAR concentration was analyzed in reference to biomarkers directly related to the cardiac structure and function—troponin T (TnT) and the N-terminal pro hormone of B-type natriuretic peptide (NT-proBNP)—and against the background of inflammation biomarker C-reactive protein (CRP) and echocardiographic images.

## 2. Materials and Methods

### 2.1. The Study Population

We recruited 320 community-dwelling consecutive individuals, patients of an outpatient geriatric clinic, aged 76–91 years (median age 80 years). Next, we excluded 82 patients who met the exclusion criteria based on knowledge of the independent factors that may significantly influence suPAR level: clinical symptoms of infection, acute inflammation (C-reactive protein >5 mg/L), end-stage renal disease (hemodialysis or stage >3a in GFR category), autoimmune diseases, immunosuppressive treatment, active malignancy and metastatic cancer. Additional exclusion criteria were atrial fibrillation or severe arrhythmias, which may affect cardiac parameters in echocardiographic examination as well as the patients’ functional or mental inability to participate in the study. The final studied group consisted of 238 individuals.

### 2.2. Determination of the Laboratory Markers and Anthropometric Parameters

Laboratory tests were performed on serum samples taken after fasting. After centrifugation, a part of the serum was aliquoted and frozen at −80 °C for up to 12 months for determining the suPAR level. Within one hour after blood collection, the following parameters were assessed with the use of standardized in vitro diagnostic assays: total cholesterol (TC), high-density lipoprotein cholesterol (HDL-C), low-density lipoprotein cholesterol (LDL-C), triglycerides (TG), creatinine (CREA), C-reactive protein (CRP), all of which were measured using the DXCAU700 analyzer, Beckman Coulter, Brea, CA, USA, while troponin T (TnT) and N-terminal pro-B type natriuretic peptide (NT-proBNP) were determined by electrochemiluminescence immunoassay with the Cobas e611 analyzer, Roche Diagnostics, Rotkreutz, Switzerland. For CRP and TnT, highly sensitive methods were applied. Glomerular filtration rate (GFR) was calculated using the Berlin Initiative Study formula [20]. For the suPAR analysis, the samples were thawed, thoroughly mixed and centrifuged, and the concentration of the protein was assessed with the use of suPARnostic ELISA assay, Virogates, Denmark, in all the samples at the same time.

Body mass index (BMI) was calculated from the anthropometric data collected with the use of RADWAG personal weight scales (WPT60 150OW; Radwag Balances and Scales, Radom, Poland).

### 2.3. Echocardiographic Assessment

Echocardiographic examination was performed on the day of the blood collection in all the patients by the same experienced cardiologist according to the current guidelines, with the use of the Vivid S70 ultrasound system (GE Medical Systems, Chicago, IL, USA, 2018) [21,22]. Three consecutive heart cycles were recorded for each view. The following parameters of cardiac structure and function were measured: interventricular septal diastolic dimension (IVSd), left ventricular diastolic and systolic dimensions (LVDd, LVSd) and posterior wall diastolic dimension (PWd). Relative wall thickness (RWT) was calculated in accordance with the formula: 2 × PWd divided by LVDd. Left ventricular mass was calculated by the Devereux formula and indexed to the body surface area (LV mass index—LVMI). LVH was diagnosed according to the criteria as LVMI > 95 g/m^2^ for women and > 115 g/m^2^ for men [21]. Left atrial (LA) assessment included antero-posterior diameter (LA diam.), LA area (LAA), and LA volume indexed by body surface area (left atrial volume index, LAVI). Left ventricular ejection fraction (LVEF) was calculated using the modified biplane Simpson’s method from the apical two- and four-chamber views. The peak early (E) and atrial (A) diastolic velocities were recorded from transmitral flow, and E/A ratio was calculated. Pulsed wave tissue Doppler imaging (TDI) was used to obtain the LV peak systolic (S’), and early diastolic (E’) mitral annular myocardial velocities from the median (septal) and lateral walls and the results were averaged. The average E/E’ ratio from septal and lateral measurements were calculated. The parameters of systolic and diastolic LV function were interpreted in accordance with the current recommendations [4,21,22].

### 2.4. Statistical Analysis

The Shapiro–Wilk test was used to assess the normality of distribution for the investigated parameters. As they were found to differ significantly from normality, the results and baseline characteristics are presented as medians with a 95% confidence interval (CI) and interquartile ranges for continuous variables, and as percentages for categorical variables. 

The analysis was performed following division into main subgroups: individuals with and without LVH, and also sex-specific where necessary. Differences in the variables between the groups were tested with the use of the Mann–Whitney U test, and Spearman’s rank correlation analysis was performed to analyze correlations between tested parameters. The occurrence of differences in categorical variables between the groups was assessed using the chi-square test. 

Multiple regression with all independent variables significant in bivariate relation-ships was performed to assess which parameters independently determine the E/E’ ratio and the presence of hypertrophy, adjusting for age and sex. After checking the model that included all the laboratory biomarkers related directly and indirectly to the cardiac structure and function, stepwise logistic regression was used to determine the best-fit model determining LVH in the studied population—this model was created taking into account also all the comorbidities considered in the study. The best-fit model was additionally assessed in sex-specific groups separately. Where necessary, variables were log transformed for the needs of multivariate analyses. The statistical analysis was performed using MedCalc Statistical Software version 17.5.5 (MedCalc Software bvba, Ostend, Belgium; https://www.medcalc.org (accessed on 10 March 2023). A significance level of α = 0.05 was used in all the tests.

## 3. Results

The baseline characteristics of the studied population are presented in Table 1. The results are also given for the main subgroups, viz. with and without LVH (LVH and non-LVH, respectively). Because the subgroups appeared not to be homogenous in terms of sex abundance (a lower proportion of men were in the non-LVH group, 11.8 vs. 39.9%, *p* < 0.0001), and because part of the laboratory biomarkers assessed in the study are sex-related, additional characteristics considering sex are presented. The typical differences described elsewhere were also evident in the studied population: women appeared to have higher TC: 5.10 (CI: 4.89–5.35) vs. 4.84 (CI: 4.30–4.98) mmol/L, *p* = 0.0008; HDL-C: 1.63 (CI: 1.58–1.68) vs. 1.37 (CI: 1.32–1.43) mmol/L, *p* < 0.0001; and suPAR: 4.02 (CI: 3.82–4.26) vs. 3.81 (CI: 3.48–3.90) ng/mL, *p* = 0.015; and lower TnT: 9.8 (CI: 8.95–10.73) vs. 14.2 (CI: 12.30–15.25) ng/L, *p* < 0.001 than the men. A higher percentage of men had a diagnosis of CAD (27.5 vs. 15%, *p* = 0.0266) and appeared to take statins (63 vs. 47%, *p* = 0.0315). At the same time, higher TC (5.20 vs. 4.86 mmol/L, *p* = 0.026) and HDL-C (1.63 vs. 1.50 mmol/L, *p* < 0.001) was visible in the non-LVH subgroup of patients, while the suPAR level appeared to be significantly higher in the LVH subgroup (4.01 vs. 3.82 ng/mL, *p* = 0.033). The patients with LVH were more likely to demonstrate HT (89 vs. 68%, *p* < 0.001), diabetes (14.7 vs. 3.1%, *p* = 0.0035) and a significantly higher glucose level (5.79 vs. 5.45 mmol/L, *p* = 0.0103). Patients with LVH turned out to have higher BMI in relation to the non-LVH subgroup (27.9 vs. 25.1 kg/m^2^, *p* < 0.001).

Table 2 presents the echocardiographic parameters of the studied population. The group with LVH was characterized by higher LV walls’ thickness and LV volumes, higher RWT and LVMI, and all the parameters describing LA. Moreover, significant differences between the groups were observed regarding E’ (lower values in the LVH group) and E/E’ ratio (higher values in the LVH group)

Taking into account the observed differences, the relationships between the described echocardiographic parameters and the biomarkers included in the study were evaluated (Table 3) with a further analysis based on the sex of the individuals. The suPAR level was significantly positively correlated with age (rS = 0.211, *p* < 0.05), E (rS = 0.151, *p* < 0.05) and E/E’ (rS = 0.249, *p* < 0.05), and negatively with E’ (rS = −0.160, *p* < 0.05) in the studied population. The observed correlations were most clearly expressed in the population of women. For the biomarkers associated with LV structure and function, viz. TnT and NT-proBNP, typical and consistent dependences were observed as described in the literature. For CRP, only the positive correlation with BMI (rS = 0.137, *p* < 0.05) was found to be statistically relevant in the studied population.

**Figure 1:**
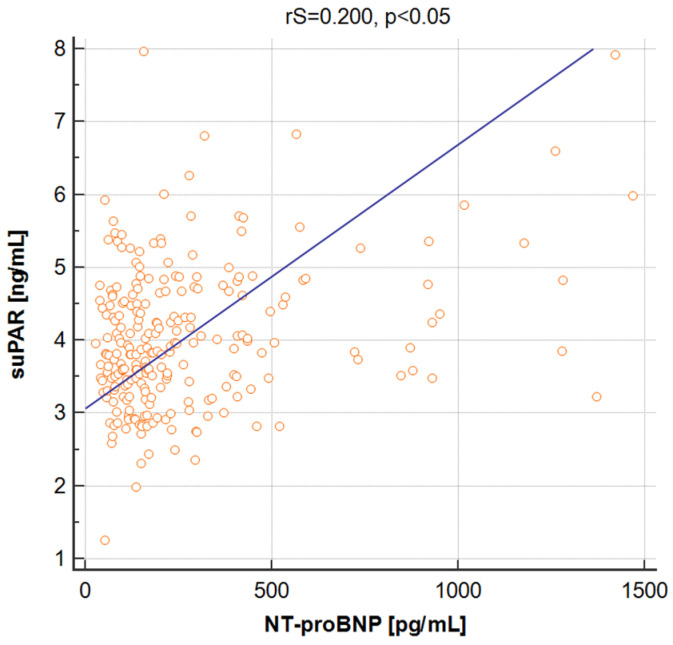
Spearman’s correlation between the suPAR and NT-proBNP levels in the studied population of patients.

The suPAR level was also related to measured biomarkers of cardiac pressure overload, cardiomyocyte damage and inflammation. Positive statistically significant correlation was found between the suPAR and NT-proBNP levels (rS = 0.200, *p* < 0.05) (Figure 1). Analogous correlations were also observed between the suPAR and TnT level (rS = 0.136, *p* < 0.05), as well as the CRP level (rS = 0.245, *p* < 0.05).

In multiple regression analysis, the suPAR level significantly determined E/E’ (ß = 0.73, *p* = 0.0001), together with the prevalence of hypertension (ß = 1.01, *p* = 0.0154); R^2^ = 0.12, while diabetes, CAD, age and sex did not enter the model. Protein level determined the occurrence of LVH independently of the other laboratory biomarkers (ß = 0.12, *p* = 0.0008), also after adjusting for sex (ß = 0.39, *p* = < 0.0001) and age (not entered into the model) (R^2^ = 0.14). The stepwise logistic regression (checked with the use of forward and backward method) found that higher suPAR level (OR = 1.58, 95%CI: 1.12–2.22, *p* = 0.009), higher BMI (OR = 1.18, 95%CI: 1.09–1.29, *p* < 0.001), prevalence of hypertension (OR = 3.00, 95%CI: 1.37–6.59, *p* = 0.006) and male sex (OR = 6.69, 95%CI: 3.05–14.68, *p* < 0.000) were associated with the occurrence of LVH, with R^2^ = 0.34 and overall fit of the model: *p* < 0.0001. As sex appeared to contribute in the relation with LVH the most, an independent analysis was performed in the sex-related subgroups; suPAR appeared to fit the model only in the group of women. The detailed characteristics of the logistic regression analysis are presented in Table 4.

## 4. Discussion

### 4.1. Clinical Characteristics of the Group

The study population consisted of community-dwelling individuals of advanced age, i.e., in the range of 77 to 91 years, of which 72% were female. In the baseline characteristics, the differences between men and women were found in the following areas: TC and HDL-C, statins intake, hsTnT and suPAR levels and comorbidities, which remains consistent with the literature [23,24,25]. The presence of LVH is considered as an indicator of subclinical cardiac damage and a prognostic factor in the general population [26,27]. Our results showed the prevalence of LVH in the study population at about 58%, with a frequency of HT of 80%. Previous studies showed that varying prevalence of LVH in different groups of patients depends on many factors, such as age, sex, obesity, HT and other comorbidities [28,29,30,31]. In terms of the clinical features, the LVH group was characterized by significantly higher BMI, fasting glucose and suPAR levels, and also lower HDL-C. This group included the majority of men and more individuals with diabetes and HT in comparison to the non-LVH group. Some of these factors are known to be related with LVH in the way observed in our study, such as sex, BMI, HT or metabolic disorders including diabetes [3,28,29,30,31,32]. An interesting new observation is the presence of a significant difference between the groups in terms of the suPAR level. 

When comparing echocardiographic parameters in both the groups, in addition to those defining hypertrophy itself, higher values of LA diameter, area and volume were observed in the LVH group. The enlargement of LA is one of the indicators of LV diastolic dysfunction, associated also with increased cardiovascular risk [22,33]. The subjects with LVH were also characterized by lower E’ and higher E/E’. The observed changes in LA, as well as those in E’ and E/E’, indicate worse LV diastolic function in this group in comparison to individuals without LVH. This observation confirms the unfavorable and age-independent direction of development of myocardial disorders on the basis of LVH, as confirmed in the literature mechanism in HF, especially in HFpEF [4].

### 4.2. TnT, NT-proBNP and CRP in Association with Cardiac Parameters

The use of natriuretic peptides as well as troponins is well established in cardiology, both in the diagnostic and prognostic context [4,34,35]. Their relationships with the parameters of cardiac structure and function observed in our study confirm those from the literature [8,36,37,38]. It is worth noticing that they also showed up in individuals of advanced age with echocardiographic parameters remaining, for the most part, still within the normal range. 

Although there was no difference regarding the TnT levels between groups with and without LVH, a significant positive correlation with the parameters describing the size of LA and negative correlation with EF was observed in both the sexes. Additionally, there was a relationship with E/E’, but in women only. When analyzing the whole studied population, significant correlations with LV wall thickness, LV volumes and LVMI were noticed. According to the literature, troponins are released secondarily as a result of myocardium damage, both in terms of its systolic and diastolic disorders [4,34,35]. Absolute greater heart muscle mass may also contribute to higher serum troponins concentration; therefore, their higher levels may appear in men [8,39,40].

NT-proBNP reflects the volume and/or pressure overload of the myocardium. In our study, the peptide’s levels did not significantly differ between the LVH and non-LVH groups. However, depending on the populations studied, it may also be elevated in individuals with LVH [8,36,40]. Confirmed in the literature is a negative correlation with EF and positive with LA measurements, as observed also in our study in both the sexes [8,36,40,41,42]. In addition, a relationship between NT-proBNP and E/E’ or LVSd was clearly marked in our population only in women. Regarding the other parameters, there also was an association between NT-proBNP levels and E/A, but its direction may suggest the impact of E/A pseudo-normalization on the results obtained. 

Similar to the above biomarkers, no differences were observed between the LVH and non-LVH groups in terms of CRP levels. Of the analyzed echocardiographic parameters, one significant correlation with IVSd was observed in women only. This may confirm the prognostic role of CRP emphasized in the literature, but not a direct and clinically significant relationship with echocardiographic parameters [42,43,44,45].

### 4.3. suPAR and Cardiac Parameters of the Elderly with and without LVH

The soluble urokinase-type plasminogen activator receptor is a novel biomarker, which has recently been widely studied in the area of cardiovascular diseases, mainly in the diagnostic and prognostic context. It reflects new pathological pathways, including immune activation, chronic inflammation and fibrosis, which may underlie cardiac damage [9,15,17,18].The important role of suPAR as prognostic marker of adverse events and disease severity in patients with CAD, HF, arrythmias or metabolic disorders at high cardiovascular risk has been confirmed [44,45,46,47,48]. 

There is a lack of studies in the literature on the potential use of suPAR level measurement in the determination of LVH, especially in the elderly, among which hypertrophy may have both physiological and pathological causes. In our study, conducted in individuals of advanced age, higher serum suPAR in the LVH group in comparison to the non-LVH group was observed, along with the absence of such differences for other biomarkers. Of the parameters of cardiac structure, suPAR correlated positively with LVSd and PWd, but not directly with LVMI. In addition, these significant relationships were shown in women only. To compare, in the study of Fujita et al., among 242 patients admitted to the cardiology department, with a mean age 71.3 ± 9.8 years (29% women), a weak correlation between suPAR level and LVMI was demonstrated (rS = 0.16, *p* = 0.014), but ultimately the authors concluded that there was no relationship between cardiac hypertrophy and suPAR in this group [49]. In another study by these authors, regarding only individuals with preserved EF (≥50%), the observed correlation between suPAR and LVMI was analogous (rS = 0.14, *p* = 0.015) but insignificant when the population was divided according to GFR [50]. Our further analysis showed that suPAR level, BMI and the presence of HT were the factors independently associated with LVH in women. In men, the strongest predictors of LVH were HT (OR = 7.52, *p* = 0.014) and BMI (OR = 1.42, *p* = 0.032). 

The differences in the epidemiology of CVD according to sex are well-documented, especially in the context of CAD and also HF, where more than 50% of patients with HFpEF are women [1,2,4,5,7]. Moreover, LVH is more frequent in hypertensive women than in men, independently of blood pressure control or antihypertensive treatment [51]. The mechanisms underlying the sex-related cardiac disorders are not fully understood, and suPAR may be promising marker in that context. An additional interesting observation in our research is the positive relationship between suPAR and the thickness of the LV posterior wall in women, which requires additional studies to be clarified. However, it is worth noting that typical, physiological, age-related cardiac remodeling concerns, to the greatest extent, the interventricular septum, known as “sigmoid septum” in the elderly [52]. 

Epidemiologically, the incidence of diastolic dysfunction is actually more common than systolic, with its particularly frequent prevalence in the elderly [1,2,4]. With regard to the parameters describing diastolic function of LV, our results showed a significant association of higher suPAR levels with worser values of E’ and E/E’, also observed only in women. The strongest relationship was found with E/E’. There are few studies available in the literature on this topic. In the first research conducted among patients with type 1 diabetes with no history of cardiac diseases, higher suPAR levels were associated with A’ and E’/A’, whose values indicated an early stage of diastolic function impairment [53]. 

Fujisaka et al. found that individuals with the presence of diastolic dysfunction have higher suPAR concentration, with a correlation coefficient with E/E’ for the whole population (rS= 0.29 (*p* < 0.001)), and a lack of relationship with E/A [50]. In conclusion, the authors indicated that suPAR levels were associated with diastolic dysfunction independently on age, sex, systolic blood pressure, renal function, CRP and diuretic treatment. Another interesting study concerned patients with lung cancer who underwent a detailed echocardiographic examination to assess early cardiac dysfunction with regard to suPAR measurements in its identification [54]. It has been shown that suPAR levels correlated significantly with E/E’, but not with E/A, which confirms our results. An additional observation was the lack of relationship between suPAR level and LVEF, as in our own and other authors’ studies, where patients with generally preserved LVEF were analyzed [50,53,54]. A significant but weak negative correlation was found earlier in the population, which also included patients with reduced LVEF [49]. In the already cited studies, an association was also found in relation to the LV systolic function in individuals with normal LVEF with S’ and global longitudinal strain (GLS) parameters [53,54]. 

To summarize, the results of our own and previously published observations indicate suPAR as a promising marker of early-stage cardiac damage, corresponding to subclinical organ changes. The mechanisms that the marker reflects may be related to chronic immune and inflammatory activation, fibrosis, vascular sclerosis and microcirculation disorders, thus indicating the complexity of the pathways underlying cardiac dysfunction [47,55].

## 5. Study Strength and Limitations

A key strength of our study is that it is based on a population of an advanced age, with echocardiographic parameters of cardiac function remaining, generally, in the normal ranges, or only slightly exceeding them. Thus, it was possible to observe the relationships that are mostly not found in the individuals with significant cardiac damage. One limitation of our study is its cross-sectional character. Additionally, echocardiographic examination did not include assessment of global longitudinal strain (GLS)—a more sensitive indicator of myocardial function. Moreover, the studied population was not balanced with regard to sex; however, this proportion reflects the demographic situation in the elderly population in Poland, where the study was performed.

## 6. Conclusions

Despite the progress in management of CVD, HF is still a huge epidemiological, social and economic problem. The increasing prevalence of HFpEF, which is related mainly to cardiac hypertrophy and diastolic dysfunction, is a predominant problem, especially in women and the elderly. Our study, conducted among individuals of an advanced age, concerned the role of the biomarker of chronic inflammation and fibrosis, suPAR, in the context of LVH and concomitant cardiac dysfunction. Correlations and stepwise logistic regression analysis showed that suPAR level is an independent predictor of LVH, especially in women, and additionally that it is associated with worse LV diastolic function. These relationships were not observed in relation to the male sex. Our results indicate the potential mechanisms underlying cardiac damage in women of an advanced age, independent of aging-associated cardiac remodeling. Further studies are needed to explain the longitudinal changes in suPAR levels in association with LV remodeling.

## Figures and Tables

**Table 1:** The baseline characteristics of the studied population divided by sex and into subgroups with and without left ventricular hypertrophy.

Variable	All *n* = 238	Men *n* = 66	Women *n* = 172	*p*	LVH, *n* = 139	Non-LVH, *n* = 99	*p*
Sex (male) *n* (%)	66 (27.7)	-	-	-	55 (39.9)	11 (11.8)	<0.001
Age [years]	80 (78–83)	80 (78–83)	80 (78–84)	ns.	80 (78–84)	80 (78–83)	ns.
BMI [kg/m^2^]	26.7 (24.3–29.8)	26.7 (25.0–29.1)	26.6 (24.0–30.3)	ns.	27.9 (25.6–30.5)	25.1 (23.2–28.7)	<0.001
TC [mmol/L]	4.94 (4.42–6.05)	4.83 (3.96–5.41)	5.09 (4.55–3.70)	<0.001	4.86 (4.29–5.74)	5.20 (4.55–6.21)	0.026
HDL-C [mmol/L]	1.55 (1.37–1.78)	1.37 (1.16–1.55)	1.63 (1.45–1.86)	<0.001	1.50 (1.32–1.73)	1.63 (1.47–1.89)	<0.001
LDL-C [mmol/L]	2.82 (2.22–3.65)	2.74 (1.94–3.57)	2.87 (2.35–3.80)	ns.	2.72 (2.17–3.47)	3.00 (2.41–3.85)	ns.
TG [mmol/L]	1.23 (0.98–1.59)	1.20 (0.97–1.57)	1.26 (0.99–1.66)	ns.	1.25 (0.99–1.51)	1.21 (0.96–1.57)	ns.
Glucose [mmol/L]	5.67 (5.22–6.40)	5.79 (5.39–6.46)	5.62 (5.22–6.35)	ns.	5.79 (5.39–6.57)	5.45 (5.11–6.29)	0.010
hsCRP [mg/L]	1.44 (0.89–2.86)	1.16 (0.69–2.65)	1.68 (0.98–2.88)	ns.	1.46 (0.89–3.27)	1.39 (0.90–2.64)	ns.
NT-proBNP [pg/mL]	170 (111–329)	161 (113–267)	182 (110–350)	ns.	171 (110–376)	170 (116–297)	ns.
hsTnT [ng/L]	10.8 (7.8–14.9)	14.2 (10.1–18.8)	9.8 (7.2–13.3)	<0.001	11.7 (7.9–15.6)	9.8 (7.7–13.5)	ns.
suPAR [ng/mL]	3.90 (3.39–4.63)	3.81 (3.29–4.17)	4.02 (3.48–4.73)	0.014	4.01 (3.48–4.71)	3.82 (3.22–4.44)	0.033
eGFR BIS [mL/min/1.72 m^2^]	56 (51–64)	59 (52–66)	56 (50–63)	ns.	57 (51–64)	56 (51–63)	ns.
Hypertension, *n* (%)	192 (80.6)	56 (84.1)	136 (79.3)	ns.	124 (89.0)	68 (68.6)	<0.001
Diabetes, *n* (%)	24 (9.9)	11 (16.9)	13 (7.8)	ns.	21 (15.1)	3 (3.1)	0.003
CAD, *n* (%)	44 (18.5)	18 (27.5)	26 (15.0)	0.026	28 (20.0)	16 (15.7)	ns.
Diuretics, *n* (%)	87 (36.7)	22 (33.8)	65 (37.9)	ns.	51 (36.8)	36 (36.6)	ns.
Statins, *n* (%)	123 (51.6)	42 (63.1)	84 (47.4)	0.0315	79 (56.7)	44 (44.4)	ns.

Abbreviations: LVH, left ventricular hypertrophy; BMI, body mass index; TC, total cholesterol; HDL-C, high-density lipoprotein cholesterol; LDL-C, low-density lipoprotein cholesterol; TG, triglycerides; hsCRP, (highly sensitive) C-reactive protein; NT-proBNP, N-terminal pro-B type natriuretic peptide; hsTnT, (highly sensitive) troponin T; suPAR, soluble urokinase-type plasminogen activator receptor; eGFR BIS, estimated glomerular filtration rate (Berlin Initiative Study); CAD, coronary artery disease; ns, not significant. The continuous variables are presented as medians with interquartile ranges, and categorical variables are presented as numbers and percentages.

**Table 2:** The echocardiographic characteristic of the studied population divided into subgroups with and without left ventricular hypertrophy.

Variable	All *n* = 238	LVH, *n* = 139	Non-LVH, *n* = 99	*p*
IVSd [cm]	1.1 (1.0–1.2)	1.1 (0.9–1.2)	1.0 (0.8–1.1)	<0.001
LVDd [cm]	4.6 (4.4–4.9)	4.7 (4.5–5.0)	4.5 (4.3–4.8)	<0.001
LVSd [cm]	2.7 (2.5–3.0)	2.8 (2.5–3.0)	2.6 (2.3–2.29)	<0.001
PWd [cm]	1.0 (0.9–1.1)	1.0 (1.0–1.0)	0.9 (0.8–1.0)	<0.001
RWT	0.44 (0.42–0.48)	0.46 (0.43–0.49)	0.42 (0.40–0.44)	<0.001
LA diam. [cm]	3.7 (3.4–4.0)	3.9 (3.5–4.1)	3.5 (3.2–3.7)	<0.001
LAA [cm^2^]	17.0 (15.0–19.5)	17.5 (15.0–20.4)	15.0 (13.0–17.0)	<0.001
LAVI [mL/m^2^]	27.0 (22.2–32.4)	29.2 (23.4–91.8)	24.7(20.5–30.2)	0.001
LVMI [g/m^2^]	110 (99–128)	121 (109–135)	99 (89–108)	<0.001
EF [%]	60 (58–62)	60 (58–62)	60 (58–63)	ns.
S’ [m/s]	0.08 (0.09–0.09)	0.08 (0.08–0.09)	0.08 (0.08–0.09)	ns.
E/A	0.70 (0.58–0.81)	0.68 (0.57–0.79)	0.72 (0.6–0.82)	ns.
E’ [m/s]	0.07 (0.06–0.08)	0.07 (0.06–0.08)	0.07 (0.06–0.09)	0.003
E/E’	8.57 (7.28–10.1)	8.9 (7.8–10.4)	8.1 (6.9–9.6)	0.003

Abbreviations: IVSd, interventricular septal diastolic dimension; LVDd, left ventricular diastolic dimension; LVSd, left ventricular systolic dimension, PWd, posterior wall diastolic dimension; RWT, relative wall thickness; LA diam., left atrial anteroposterior diameter; LAA, left atrial area; LAVI, left atrial volume index; LVMI, left ventricular mass index; LVEF, left ventricular ejection fraction; S’, average systolic mitral annular velocity; E/A, ratio of early to late mitral inflow velocities; E’, average early diastolic mitral annular velocity; E/E’, an average ratio of early mitral diastolic inflow velocity to early diastolic mitral annular velocity; LVH, left ventricular hypertrophy; ns., not significant. The variables are presented as medians with interquartile ranges.

**Table 3:** Spearman’s correlations of serum biomarkers with echocardiographic parameters in the studied population, also divided into subgroups by sex.

Variable	suPAR [ng/mL]			hsTnT [ng/L]			NT-proBNP [pg/mL]			hsCRP [mg/L]		
	All	Men	Women	All	Men	Women	All	Men	Women	All	Men	Women
Age [years]	0.212 *	0.087	0.268 *	0.335 *	0.301	0.371	0.273 *	0.248 *	0.283 *	−0.006	0.034	−0.015
BMI [kg/m^2^]	0.088	−0.052	0.138	0.034	−0.039	0.055	−0.158 *	−0.110	−0170 *	0.137 *	0.210	0.125
IVSd [cm]	0.066	0.147	0.137	0.200 *	0.234	0.033	0.033	0.183	0.009	0.063	0.029	0.173 *
LVDd [cm]	−0.009	−0.023	0.096	0.154 *	0.204	0.006	0.026	0.167	0.074	−0.023	0.104	0.031
LVSd [cm]	0.078	−0.033	0.216 *	0.213 *	0.120	0.135	0.156 *	0.186	0.225 *	0.018	0.084	0.078
PWd [cm]	0.058	0.040	0.169 *	0.231 *	0.214	0.101	0.067	0.191	0.063	0.022	−0.035	0.119
RWT	0.052	0.117	0.042	0.060	0.113	0.047	−0.016	0.078	−0.043	0.046	−0.105	0.111
LA diam [cm]	0.077	0.186	0.135	0.296 *	0.314 *	0.176 *	0.186 *	0.251 *	0.227 *	0.004	0.130	0.047
LAA [cm^2^]	0.061	0.110	0.130	0.364 *	0.400 *	0.279 *	0.250 *	0.292 *	0.290 *	−0.047	0.107	−0.031
LAVI [mL/m^2^]	0.040	0.014	0.099	0.331 *	0.330 *	0.293 *	0.321 *	0.370 *	0.337 *	−0.043	0.036	−0.048
LVMI [g/m^2^]	0.021	−0.016	0.134	0.132 *	0.153	0.011	0.071	0.205	0.084	−0.014	−0.055	0.078
EF [%]	0.021	0.126	−0.029	−0.169 *	−0.272 *	−0.159 *	−0.219 *	−0.362 *	−0.168 *	−0.018	−0.084	−0.018
S’ [m/s]	−0.010	−0.025	0.020	0.082	−0.205	0.133	−0.184 *	−0.321 *	−0.118	−0.015	−0.134	0.040
E/A	0.106	0.124	0.098	0.045	0.073	0.081	0.350 *	0.244 *	0.377 *	0.052	0.071	0.041
E’ [m/s]	−0.160 *	0.034	−0.217 *	−0.004	−0.003	−0.011	0.031	−0.034	0.060	−0.021	0.089	−0.073
E/E’	0.249 *	−0.032	0.342 *	0.085	0.025	0.186 *	0.186 *	0.186	0.174 *	0.083	0.016	0.107

* *p* < 0.05. Abbreviations: suPAR, soluble urokinase-type plasminogen activator receptor; BMI, body mass index; hsTnT (highly sensitive) troponin T; NT-proBNP, N-terminal pro-B type natriuretic peptide; hsCRP, (highly sensitive) C-reactive protein; IVSd, interventricular septal diastolic dimension; LVDd, left ventricular diastolic dimension; LVSd, left ventricular systolic dimension, PWd, posterior wall diastolic dimension; RWT, relative wall thickness; LA diam, left atrial anteroposterior diameter; LAA, left atrial area; LAVI, left atrial volume index; LVMI, left ventricular mass index; LVEF, left ventricular ejection fraction; S’, average systolic mitral annular velocity; E/A, ratio of early to late mitral inflow velocities; E’, average early diastolic mitral annular velocity; E/E’, an average ratio of early mitral diastolic inflow velocity to early diastolic mitral annular velocity; LV, left ventricle.

**Table 4:** Statistical characteristics of the stepwise logistic regression analysis of the best-fit general model for determining the occurrence of LVH in the studied population and sex-related subgroups.

Variables	All Patients OR (95%CI), *n* = 238	Women OR (95%CI), *n* = 172	Men OR (95%CI) *n* = 66
Male sex	6.69 (3.05–14.68) ***	-	-
suPAR	1.57 (1.12–2.22) **	1.55 (1.08–2.23) *	not fit into the model
BMI	1.19 (1.09–1.29) ***	1.17 (1.07–1.27) ***	1.42 (1.03–1.96) *
Hypertension	3.00 (1.37–6.59) **	2.42 (1.02–5.79) *	7.52 (1.50–37.50) *
Overall fit of the model; *p*	<0.0001	<0.0001	0.0009

* *p* < 0,05; ** *p* < 0.01; *** *p* < 0.001. Abbreviations: suPAR, soluble urokinase-type plasminogen activator receptor; BMI, body mass index.

## Data Availability

Available on demand.

## References

[1] van Riet E.E., Hoes A.W., Wagenaar K.P., Limburg A., Landman M.A., Rutten F.H. (2016). Epidemiology of heart failure: The prevalence of heart failure and ventricular dysfunction in older adults over time. A systematic review. Eur. J. Heart Fail..

[2] Virani S.S., Alonso A., Benjamin E.J., Bittencourt M.S., Callaway C.W., Carson A.P., Chamberlain A.M., Chang A.R., Cheng S., Delling F.N. (2020). Heart Disease and Stroke Statistics—2020 Update: A Report from the American Heart Association. Circulation.

[3] Cuspidi C., Sala C., Negri F., Mancia G., Morganti A., Italian Society of Hypertension (2011). Prevalence of left-ventricular hypertrophy in hypertension: An updated review of echocardiographic studies. J. Hum. Hypertens..

[4] McDonagh T.A., Metra M., Adamo M., Gardner R.S., Baumbach A., Böhm M., Burri H., Butler J., Čelutkienė J., Chioncel O. (2021). 2021 ESC Guidelines for the diagnosis and treatment of acute and chronic heart failure. Eur. Heart J..

[5] Gebhard C., Stähli B.E., Gebhard C.E., Tasnady H., Zihler D., Wischnewsky M.B., Jenni R., Tanner F.C. (2013). Age- and Gender-Dependent Left Ventricular Remodeling. Echocardiography.

[6] Gong F.F., Coller J.M., McGrady M., Boffa U., Shiel L., Liew D., Stewart S., Owen A.J., Krum H., Reid C.M. (2020). Age-related longitudinal change in cardiac structure and function in adults at increased cardiovascular risk. ESC Heart Fail..

[7] Merz A., Cheng S. (2016). Sex differences in cardiovascular ageing. Heart.

[8] Eggers K.M., Lindahl B., Venge P., Lind L. (2018). Predictors of 10-year changes in levels of N-terminal pro B-type natriuretic peptide and cardiac troponin I in the elderly. Int. J. Cardiol..

[9] Rasmussen L.J.H., Petersen J.E.V., Eugen-Olsen J. (2021). Soluble Urokinase Plasminogen Activator Receptor (suPAR) as a Biomarker of Systemic Chronic Inflammation. Front. Immunol..

[10] Backes Y., Van Der Sluijs K.F., Mackie D.P., Tacke F., Koch A., Tenhunen J.J., Schultz M.J. (2012). Usefulness of suPAR as a biological marker in patients with systemic inflammation or infection: A systematic review. Intensiv. Care Med..

[11] Velissaris D., Zareifopoulos N., Karamouzos V., Pierrakos C., Karanikolas M. (2022). Soluble urokinase plasminogen activator receptor (suPAR) in the emergency department: An update. Casp. J. Intern. Med..

[12] Guthoff M., Wagner R., Randrianarisoa E., Hatziagelaki E., Peter A., Häring H.-U., Fritsche A., Heyne N. (2017). Soluble urokinase receptor (suPAR) predicts microalbuminuria in patients at risk for type 2 diabetes mellitus. Sci. Rep..

[13] Hayek S.S., Sever S., Ko Y.-A., Trachtman H., Awad M., Wadhwani S., Altintas M.M., Wei C., Hotton A.L., French A.L. (2015). Soluble Urokinase Receptor and Chronic Kidney Disease. N. Engl. J. Med..

[14] Meijers B., Poesen R., Claes K., Dietrich R., Bammens B., Sprangers B., Naesens M., Storr M., Kuypers D., Evenepoel P. (2015). Soluble urokinase receptor is a biomarker of cardiovascular disease in chronic kidney disease. Kidney Int..

[15] Pemberton C. (2017). Prognostic Outcomes in Patients with Heart Failure: A New SuPAR Biomarker for Risk Prediction?. JACC: Heart Fail..

[16] Thunø M., Macho B., Eugen-Olsen J. (2009). suPAR: The molecular crystal ball. Dis. Markers.

[17] Goodchild T.T., Li Z., Lefer D.J. (2022). Soluble urokinase plasminogen activator receptor: From biomarker to active participant in atherosclerosis and cardiovascular disease. J. Clin. Investig..

[18] Velissaris D., Zareifopoulos N., Koniari I., Karamouzos V., Bousis D., Gerakaris A., Platanaki C., Kounis N. (2021). Soluble Urokinase Plasminogen Activator Receptor as a Diagnostic and Prognostic Biomarker in Cardiac Disease. J. Clin. Med. Res..

[19] Nikorowitsch J., Borchardt T., Appelbaum S., Ojeda F., Lackner K.J., Schnabel R.B., Blankenberg S., Zeller T., Karakas M. (2020). Cardio-Renal Biomarker Soluble Urokinase-Type Plasminogen Activator Receptor Is Associated with Cardiovascular Death and Myocardial Infarction in Patients with Coronary Artery Disease Independent of Troponin, C-Reactive Protein, and Renal Function. J. Am. Heart Assoc..

[20] Schaeffner E.S., Ebert N., Delanaye P., Frei U., Gaedeke J., Jakob O., Kuhlmann M.K., Schuchardt M., Tölle M., Ziebig R. (2012). Two Novel Equations to Estimate Kidney Function in Persons Aged 70 Years or Older. Ann. Intern. Med..

[21] Lang R.M., Badano L.P., Mor-Avi V., Afilalo J., Armstrong A., Ernande L., Flachskampf F.A., Foster E., Goldstein S.A., Kuznetsova T. (2015). Recommendations for Cardiac Chamber Quantification by Echocardiography in Adults: An Update from the American Society of Echocardiography and the European Association of Cardiovascular Imaging. J. Am. Soc. Echocardiogr..

[22] Nagueh S.F., Smiseth O.A., Appleton C.P., Byrd B.F., Dokainish H., Edvardsen T., Flachskampf F.A., Gillebert T.C., Klein A.L., Lancellotti P. (2016). Recommendations for the Evaluation of Left Ventricular Diastolic Function by Echocardiography: An Update from the American Society of Echocardiography and the European Association of Cardiovascular Imaging. J. Am. Soc. Echocardiogr..

[23] Wlazel R.N., Szwabe K., Guligowska A., Kostka T. (2020). Soluble urokinase plasminogen activator receptor level in individuals of advanced age. Sci. Rep..

[24] Lam C.S.P., Arnott C., Beale A.L., Chandramouli C., Hilfiker-Kleiner D., Kaye D.M., Ky B., Santema B.T., Sliwa K., A Voors A. (2019). Sex differences in heart failure. Eur. Heart J..

[25] Tromp J., A Paniagua S.M., Lau E.S., Allen N.B., Blaha M.J., Gansevoort R.T., Hillege H.L., E Lee D., Levy D., Vasan R.S. (2021). Age dependent associations of risk factors with heart failure: Pooled population based cohort study. BMJ.

[26] Williams B., Mancia G., Spiering W., Agabiti Rosei E., Azizi M., Burnier M., Clement D.L., Coca A., de Simone G., Dominiczak A. (2018). 2018 ESC/ESH Guidelines for the management of arterial hypertension. Eur. Heart J..

[27] Giamouzis G., Dimos A., Xanthopoulos A., Skoularigis J., Triposkiadis F. (2021). Left ventricular hypertrophy and sudden cardiac death. Heart Fail. Rev..

[28] Ruilope L.M., Schmieder R.E. (2008). Left Ventricular Hypertrophy and Clinical Outcomes in Hypertensive Patients. Am. J. Hypertens..

[29] Cheng S., Xanthakis V., Sullivan L.M., Lieb W., Massaro J., Aragam J., Benjamin E.J., Vasan R.S. (2010). Correlates of echocardiographic indices of cardiac remodeling over the adult life course: Longitudinal observations from the Framingham Heart Study. Circulation.

[30] Cuspidi C., Rescaldani M., Sala C., Grassi G. (2014). Left-ventricular hypertrophy and obesity: A systematic review and meta-analysis of echocardiographic studies. J. Hypertens..

[31] Tong M., Saito T., Zhai P., Oka S.-I., Mizushima W., Nakamura M., Ikeda S., Shirakabe A., Sadoshima J. (2019). Mitophagy Is Essential for Maintaining Cardiac Function During High Fat Diet-Induced Diabetic Cardiomyopathy. Circ. Res..

[32] Succurro E., Miceli S., Fiorentino T.V., Sciacqua A., Perticone M., Andreozzi F., Sesti G. (2021). Sex-specific differences in left ventricular mass and myocardial energetic efficiency in non-diabetic, pre-diabetic and newly diagnosed type 2 diabetic subjects. Cardiovasc. Diabetol..

[33] Cuspidi C., Rescaldani M., Sala C. (2013). Prevalence of Echocardiographic Left-Atrial Enlargement in Hypertension: A Systematic Review of Recent Clinical Studies. Am. J. Hypertens..

[34] Ibanez B., James S., Agewall S., Antunes M.J., Bucciarelli-Ducci C., Bueno H., Caforio A.L.P., Crea F., Goudevenos J.A., Halvorsen S. (2018). 2017 ESC Guidelines for the management of acute myocardial infarction in patients presenting with ST-segment elevation: The Task Force for the management of acute myocardial infarction in patients presenting with ST-segment elevation of the European Society of Cardiology (ESC). Eur. Heart J..

[35] Collet J.-P., Thiele H., Barbato E., Barthélémy O., Bauersachs J., Bhatt D.L., Dendale P., Dorobantu M., Edvardsen T., Folliguet T. (2021). 2020 ESC Guidelines for the management of acute coronary syndromes in patients presenting without persistent ST-segment elevation. Eur. Heart J..

[36] Bielecka-Dabrowa A., Michalska-Kasiczak M., Gluba A., Ahmed A., Gerdts E., von Haehling S., Rysz J., Banach M. (2015). Biomarkers and Echocardiographic Predictors of Myocardial Dysfunction in Patients with Hypertension. Sci. Rep..

[37] Suthahar N., Lau E.S., Blaha M.J., Paniagua S.M., Larson M.G., Psaty B.M., Benjamin E.J., Allison M.A., Bartz T.M., Januzzi J.L. (2020). Sex-Specific Associations of Cardiovascular Risk Factors and Biomarkers with Incident Heart Failure. J. Am. Coll. Cardiol..

[38] Lind L., Loader J., Lindahl B., Eggers K.M., Sundström J. (2022). A comparison of echocardiographic and circulating cardiac biomarkers for predicting incident cardiovascular disease. PLoS ONE.

[39] Forghani M.S., Jadidoleslami M.S., Naleini S.N., Rajabnia M. (2018). Measurement of the serum levels of serum troponins I and T, albumin and C-Reactive protein in chronic hemodialysis patients and their relationship with left ventricular hypertrophy and heart failure. Diabetes Metab. Syndr. Clin. Res. Rev..

[40] Le T.-T., Lim V., Ibrahim R., Teo M.-T., Bryant J., Ang B., Su B., Aw T.-C., Lee C.-H., Bax J. (2020). The remodelling index risk stratifies patients with hypertensive left ventricular hypertrophy. Eur. Heart J.-Cardiovasc. Imaging.

[41] Gehlken C., Screever E.M., Suthahar N., van der Meer P., Westenbrink B.D., Coster J.E., Van Veldhuisen D.J., de Boer R.A., Meijers W.C. (2021). Left atrial volume and left ventricular mass indices in heart failure with preserved and reduced ejection fraction. ESC Heart Fail..

[42] Wang Q., An Y., Wang H., Zhang N., Deng S. (2021). The clinical significance of changes in cTnT, CRP and NT-proBNP levels in patients with heart failure. Am. J. Transl. Res..

[43] Lima P.C., Rios D.M., de Oliveira F.P., Passos L.R., Ribeiro L.B., Serpa R.G., Calil O.A., de Barros L.C., Barbosa L.F.M., Barbosa R.R. (2022). Inflammation as a Prognostic Marker in Heart Failure. Cureus.

[44] Sørensen M.H., Gerke O., Eugen-Olsen J., Munkholm H., Lambrechtsen J., Sand N.P.R., Mickley H., Rasmussen L.M., Olsen M.H., Diederichsen A. (2014). Soluble urokinase plasminogen activator receptor is in contrast to high-sensitive C-reactive-protein associated with coronary artery calcifications in healthy middle-aged subjects. Atherosclerosis.

[45] Hayek S.S., Divers J., Raad M., Xu J., Bowden D.W., Tracy M., Reiser J., Freedman B.I. (2018). Predicting Mortality in African Americans with Type 2 Diabetes Mellitus: Soluble Urokinase Plasminogen Activator Receptor, Coronary Artery Calcium, and High-Sensitivity C-Reactive Protein. J. Am. Heart Assoc..

[46] Eapen D.J., Manocha P., Ghasemzadeh N., Patel R.S., Al Kassem H., Hammadah M., Veledar E., Le N., Pielak T., Thorball C.W. (2014). Soluble Urokinase Plasminogen Activator Receptor Level Is an Independent Predictor of the Presence and Severity of Coronary Artery Disease and of Future Adverse Events. J. Am. Heart Assoc..

[47] Mekonnen G., Corban M.T., Hung O.Y., Eshtehardi P., Eapen D.J., Al-Kassem H., Rasoul-Arzrumly E., Gogas B.D., McDaniel M.C., Pielak T. (2015). Plasma soluble urokinase-type plasminogen activator receptor level is independently associated with coronary microvascular function in patients with non-obstructive coronary artery disease. Atherosclerosis.

[48] Al-Badri A., Tahhan A.S., Sabbak N., Alkhoder A., Liu C., Ko Y., Vaccarino V., Martini A., Sidoti A., Goodwin C. (2020). Soluble Urokinase-Type Plasminogen Activator Receptor and High-Sensitivity Troponin Levels Predict Outcomes in Nonobstructive Coronary Artery Disease. J. Am. Heart Assoc..

[49] Fujita S.-I., Tanaka S., Maeda D., Morita H., Fujisaka T., Takeda Y., Ito T., Ishizaka N. (2017). Serum Soluble Urokinase-Type Plasminogen Activator Receptor Is Associated with Low Left Ventricular Ejection Fraction and Elevated Plasma Brain-Type Natriuretic Peptide Level. PLoS ONE.

[50] Fujisaka T., Fujita S.-I., Maeda D., Shibata K., Takahashi H., Morita H., Takeda Y., Ito T., Sohmiya K., Hoshiga M. (2017). Association between suPAR and cardiac diastolic dysfunction among patients with preserved ejection fraction. Heart Vessel..

[51] Muiesan M.L., Paini A., Aggiusti C., Bertacchini F., Rosei C.A., Salvetti M. (2018). Hypertension and Organ Damage in Women. High Blood Press. Cardiovasc. Prev..

[52] Forman D.E., de Lemos J.A., Shaw L.J., Reuben D.B., Lyubarova R., Peterson E.D., Spertus J.A., Zieman S., Salive M.E., Rich M.W. (2020). Cardiovascular Biomarkers and Imaging in Older Adults: JACC Council Perspectives. J. Am. Coll. Cardiol..

[53] Theilade S., Rossing P., Eugen-Olsen J., Jensen J.S., Jensen M.T. (2016). suPAR level is associated with myocardial impairment assessed with advanced echocardiography in patients with type 1 diabetes with normal ejection fraction and without known heart disease or end-stage renal disease. Eur. J. Endocrinol..

[54] Manshad A.S., Ballout F.A., Borgia J.A., Reiser J., Okwuosa T.M. (2022). Soluble Urokinase Plasminogen Activator Receptor Is Associated with Subclinical Myocardial Impairment by Speckle Tracking Echocardiography in Lung Cancer Patients. Front. Cardiovasc. Med..

[55] Sehestedt T., Lyngbæk S., Eugen-Olsen J., Jeppesen J., Andersen O., Hansen T., Linneberg A., Jørgensen T., Haugaard S., Olsen M. (2011). Soluble urokinase plasminogen activator receptor is associated with subclinical organ damage and cardiovascular events. Atherosclerosis.

